# FOXM1 promotes reprogramming of glucose metabolism in epithelial ovarian cancer cells via activation of GLUT1 and HK2 transcription

**DOI:** 10.18632/oncotarget.10103

**Published:** 2016-06-16

**Authors:** Yu Wang, Yuyu Yun, Bo Wu, Li Wen, Mingling Wen, Huiling Yang, Lisheng Zhao, Wenchao Liu, Suyun Huang, Ning Wen, Yu Li

**Affiliations:** ^1^ Department of Oncology, State Key Discipline of Cell Biology, Xijing Hospital, The Fourth Military Medical University, Xi'an, Shaanxi, China; ^2^ Institute of Stomatology, Chinese PLA General Hospital, Beijing, China; ^3^ State Key Laboratory of Cancer Biology, Cell Engineering Research Center & Department of Cell Biology, The Fourth Military Medical University, Xi'an, Shaanxi, China; ^4^ Department of Pharmacy, Affiliated Hospital of Academy of Military Medical Sciences, Beijing, China; ^5^ Department of Neurosurgery, The University of Texas MD Anderson Cancer Center, Houston, Texas, USA; ^6^ Program in Cancer Biology, The University of Texas Graduate School of Biomedical Sciences at Houston, Houston, Texas, USA

**Keywords:** FOXM1, GLUT1, HK2, epithelial ovarian cancer, glucose metabolism

## Abstract

Cancer cells exhibit the reprogrammed metabolism mainly via aerobic glycolysis, a phenomenon known historically as the Warburg effect; however, the underlying mechanisms remain largely unknown. In this study, we characterized the critical role of transcription factor Forkhead box protein M1 (FOXM1) in aerobic glycolysis of human epithelial ovarian cancer (EOC) and its molecular mechanisms. Our data showed that aberrant expression of FOXM1 significantly contributed to the reprogramming of glucose metabolism in EOC cells. Aerobic glycolysis and cell proliferation were down-regulated in EOC cells when FOXM1 gene expression was suppressed by RNA interference. Moreover, knockdown of FOXM1 in EOC cells significantly reduced glucose transporter 1 (GLUT1) and hexokinase 2 (HK2) expression. FOXM1 bound directly to the GLUT1 and HK2 promoter regions and regulated the promoter activities and the expression of the genes at the transcriptional level. This reveals a novel mechanism by which glucose metabolism is regulated by FOXM1. Importantly, we further demonstrated that the expression levels of FOXM1, GLUT1 and HK2 were significantly increased in human EOC tissues relative to normal ovarian tissues, and that FOXM1 expression was positively correlated with GLUT1 and HK2 expression. Taken together, our results show that FOXM1 promotes reprogramming of glucose metabolism in EOC cells via activation of GLUT1 and HK2 transcription, suggesting that FOXM1 may be an important target in aerobic glycolysis pathway for developing novel anticancer agents.

## INTRODUCTION

According to new statistics offered by the American Cancer Society, ovarian cancer is the fifth most common cause of cancer-related death among women and the most lethal gynecologic cancer in the United States [1]. In 2015, it is estimated that new cases and deaths from ovarian cancer in the United States was 21,290 and 14,180 respectively [1]. Most patients are diagnosed with an already advanced disease, and no specific biomarker is clinically available for screening and early diagnosis [2]. EOC constitutes approximately 90% of ovarian malignancies, and most patients present with widely metastatic disease at diagnosis and this results in a poor prognosis. Therefore, this necessitates a better understanding of the molecular mechanisms underlying EOC, which may play an important role in developing better early diagnostic and prognostic biomarkers.

It has been widely recognized that deregulating cellular energetics is emerging as a characteristic hallmark of cancer cells and a key contributor to tumor development [3–5]. Most cancer cells primarily utilize aerobic glycolysis for their energy needs even under normal oxygen concentrations, a phenomenon known as the Warburg effect [6]. The Warburg effect not only allows cancer cells to serve their energetic demands and provide the essential carbon and nitrogen used in macromolecule synthesis, but it also minimizes reactive oxygen species production in mitochondria, thereby fueling the rapid growth and proliferation seen in tumors [7–9]. In patients with epithelial ovarian cancer, 2-[^18^F] fluoro-2-deoxy-D-glucose (^18^F-FDG) positron emission tomography/computed tomography (PET/CT) is useful in diagnosing, staging, detecting recurrent lesions, and monitoring treatment response [10–13]. It is still not completely clear why increased glucose metabolism is selected by proliferating cancer cells. However, recent studies demonstrated that alterations in signaling pathways, which serve to increase glucose uptake, glycolysis, angiogenesis and stress resistance, may contribute to the reprogramming of glucose metabolism [14–16].

As a typical proliferation-associated transcription factor, FOXM1 mainly exerts its function in tumorigenesis through transcriptional regulation of its target genes to initiate various cellular responses, including cell growth, proliferation, differentiation, longevity and transformation [17–19]. It belongs to a large family of evolutionary conserved transcription factors that were characterized by a conserved DNA binding domain called Forkhead or winged-helix domain [19–21]. FOXM1 is frequently overexpressed in many human cancers, and its expression is associated with poor cancer outcomes [19, 22–30]. In a previous study, we found that FOXM1 was overexpressed in EOC cells and promoted EOC development and progression [31]. Furthermore, a recent study found that FOXM1 played important roles in aerobic glycolysis and tumorigenesis in patients with pancreatic cancer via transcriptional regulation of lactate dehydrogenase A (LDHA) expression [32]. However, the impact of FOXM1-mediated changes in energy dependency on human EOC progression and the mechanism underlying FOXM1-mediated glycolysis are still not fully understood.

In the present study, we demonstrated that the expression levels of FOXM1, GLUT1 and HK2 were significantly higher in EOC tissues than in normal ovarian tissues. FOXM1 expression was positively correlated with GLUT1 and HK2 expression in EOC tissues. Moreover, we found that FOXM1 could promote reprogramming of glucose metabolism by directly binding to the promoter and promoting the transcription of critical glycolytic genes GLUT1 and HK2. Therefore, our data suggest that FOXM1 is a novel transcriptional regulator of glycolysis in EOC and it may be a potential therapeutic target for treatment of patients with EOC.

## RESULTS

### Knockdown of FOXM1 downregulates GLUT1 and HK2 expression in EOC cells

**Figure 1:**
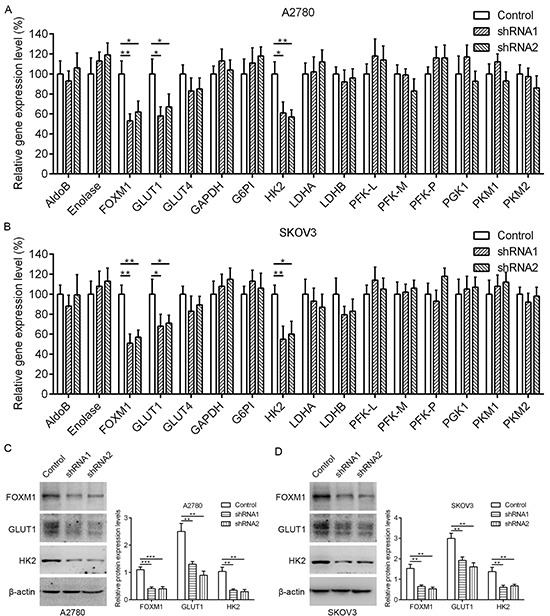
Downregulation of FOXM1 decreases GLUT1 and HK2 expression in EOC cells **A.** and **B.** qRT-PCR analysis of the mRNA expression for glycolytic enzymes in A2780 and SKOV3 cells after knockdown of FOXM1 expression. Data are shown in percentage relative to control-transfected cells. Gene expression was determined relative to β-actin. **C.** and **D.** A2780 and SKOV3 cells were either transfected with nonspecific shRNA (Control) or FOXM1 shRNA (shRNA), and analyzed by western blot analysis. The β-actin protein served as a loading control. Data represent mean ± SD from three independent replicates (*P < 0.05, **P < 0.01, ***P < 0.001 by Student's t-test).

Aerobic glycolysis is the primary aspect of metabolic reprogramming in cancer, and it is critical to the survival and proliferation of cancer cells. To determine whether FOXM1 is a key mediator of aerobic glycolysis, negative control shRNA (control) and FOXM1 shRNAs (shRNA1 and shRNA2) were transfected into A2780 and SKOV3 human EOC cell lines. We examined the effect of FOXM1 knockdown on the expression of a number of key genes involved in glycolysis, including GLUT1, GLUT4, HK2, lactate dehydrogenase isoform A (LDHA), and so on. Quantitative real-time PCR analyses showed that GLUT1 and HK2 mRNA levels were significantly decreased by FOXM1 knockdown in A2780 and SKOV3 cells (Figure 1A and 1B). In line with our above results, western blot assays showed that GLUT1 and HK2 protein levels were significantly decreased by FOXM1 knockdown in A2780 and SKOV3 cells (Figure 1C and 1D). Collectively, these results indicate that knockdown of FOXM1 downregulates GLUT1 and HK2 expression in EOC cells. Given that GLUT1 and HK2 are key metabolic enzymes involved in glycolysis, and that their expressions are dramatically regulated by FOXM1, we reasoned that FOXM1 upregulation likely plays a major role in the enhancement of glycolysis. We thus focused on the regulation of GLUT1 and HK2 for further mechanistic and functional studies.

### Knockdown of FOXM1 inhibits glycolysis in EOC cells

**Figure 2:**
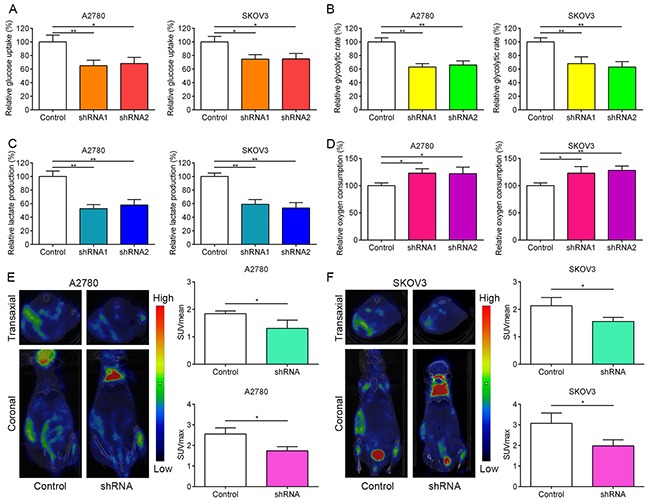
FOXM1 increases aerobic glycolysis in EOC cells **A-D.** A2780 and SKOV3 cells were transfected with FOXM1 shRNA or control shRNA. The knockdown efficiency was determined by western blot analysis. Relative glucose uptake, glycolytic rate, lactate production and oxygen consumption were measured in A2780 and SKOV3 cells transfected with control shRNA or FOXM1 shRNA. **E.** and **F.** ^18^FDG uptake in xenograft tumors with FOXM1 knockdown. Left, a representative microPET/CT image; right, Quantitative tumor ^18^FDG uptake is presented as SUVmean and SUVmax. Data are presented as mean ± SD (n = 3). *P < 0.05, **P < 0.01 by Student's t-test.

Recently, FOXM1 was found to regulate glucose metabolism in pancreatic cancer via transactivation of LDHA expression [32]. Given the importance of GLUT1 and HK2 in reprogramming of glucose metabolism in cancer cells, we hypothesized that aberrant expression of FOXM1 in EOC cells could also promote reprogramming of glucose metabolism, one of the hallmarks of cancer, to facilitate cancer proliferation. To determine whether FOXM1 regulate glucose metabolism in EOC cells, we transfected A2780 and SKOV3 cells with negative control shRNA (control) and FOXM1 shRNAs (shRNA1 and shRNA2). The results showed that glucose uptake, glycolysis rate and lactate production were significantly decreased, whereas oxygen consumption was strongly increased by FOXM1 knockdown in A2780 and SKOV3 cells (Figure 2A-2D). These results clearly show that knockdown of FOXM1 can repress the aerobic glycolysis in EOC cells, which is consistent with the previous report [32].

### Knockdown of FOXM1 inhibits ^18^F-FDG uptake and proliferation of EOC cells

To further confirm the *in vitro* phenotype of FOXM1 in glucose metabolism, we subcutaneously injected nude mice with the stable FOXM1-silenced A2780 and SKOV3 cells. We used the mean standard uptake value (SUVmean) and maximum standard uptake value SUV (SUVmax) as indexes of ^18^F-FDG accumulation. As shown in Figure 2E and 2F, micro-PET/CT imaging showed that silencing FOXM1 with shRNA led to weak ^18^F-FDG uptake compared to the control group in A2780 and SKOV3 cells.

**Figure 3:**
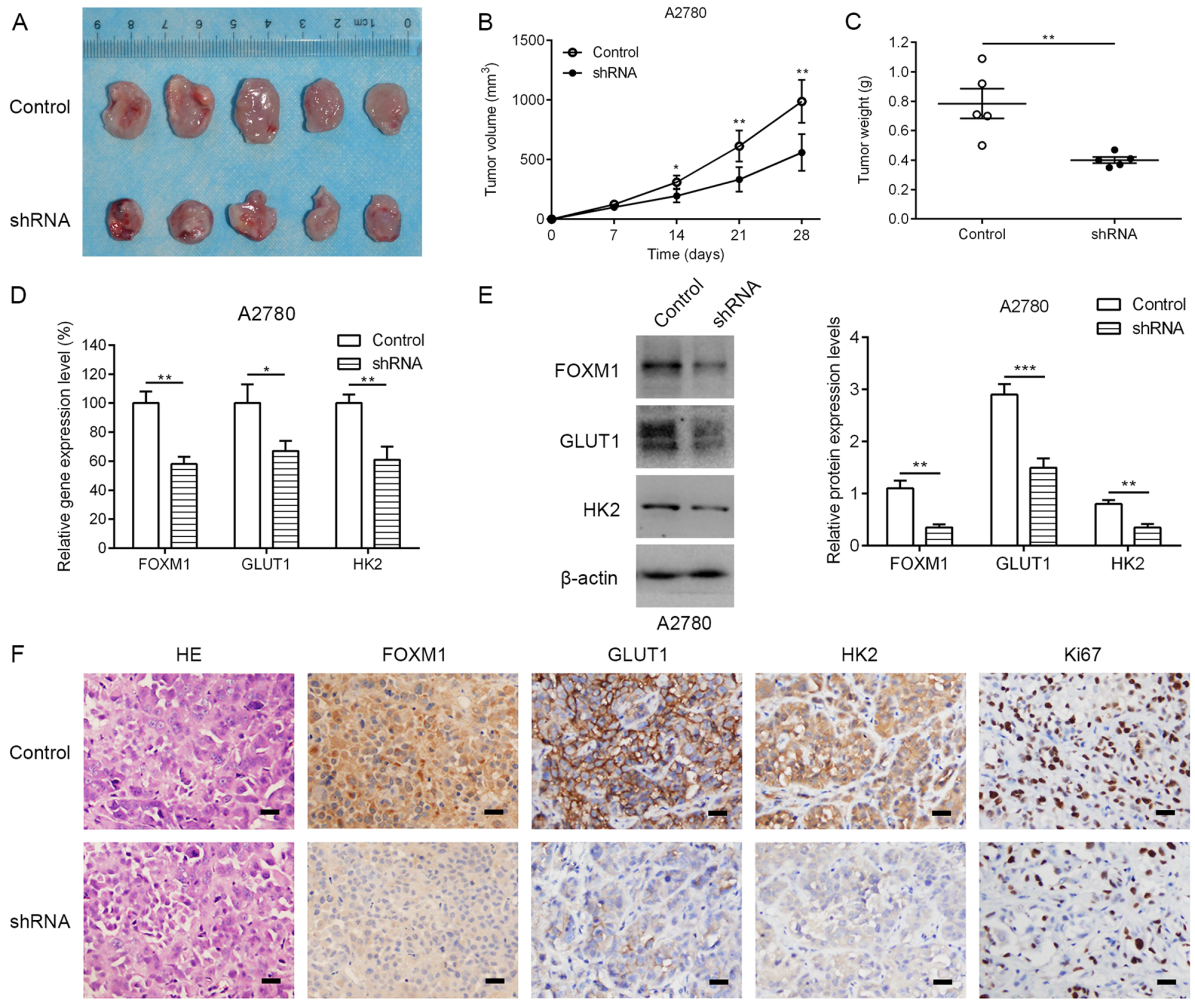
Knocking down FOXM1 expression in human EOC cells reduces tumorigenic properties **A.** representative photographs of mice from each group injected with A2780-control or A2780-shFOXM1 cells. **B.** Tumor volumes were calculated after injection every 7 days. **C.** Tumor weight derived from FOXM1-shRNA knockdown or control-shRNA knockdown was measured at day 28. **D-F.** the expression levels of FOXM1, GLUT1 and HK2 were analyzed by qRT–PCR, western blotting and immunohistochemistry. Scale bar represents 100 μm. Data are represented as means ± SD of each group. *P < 0.05, **P < 0.01, ***P < 0.001 by Student's t-test.

To determine the effect of stable loss of FOXM1 on *in vivo* subcutaneous xenografts, A2780 FOXM1-silenced cells and A2780 shRNA-control cells were injected subcutaneously into BALB/C nude mice. By 4 weeks, the smaller tumors were seen in mice injected with FOXM1-silenced cells, in contrast to shRNA-control group (Figure 3A). Compared with shRNA-control group, FOXM1-silenced tumors had a decreased proliferative index and a significant reduction in tumor weight (Figure 3B and 3C). Western blot and qRT-PCR analyses showed that the expression of GLUT1 and HK2 was decreased by FOXM1 knockdown, which was further confirmed by immunohistochemical examination of xenograft tumor sections (Figure 3D-3F). Immunohistochemical analysis also showed that the cell proliferation marker Ki67 was downregulated in A2780 cells by FOXM1 knockdown. Since GLUT1 and HK2 are critical enzymes involved in reprogramming of glucose metabolism in cancer cells, we next sought to determine whether GLUT1 and HK2 are directly regulated by FOXM1 in EOC cells.

### FOXM1 is a transcriptional activator of GLUT1

**Figure 4:**
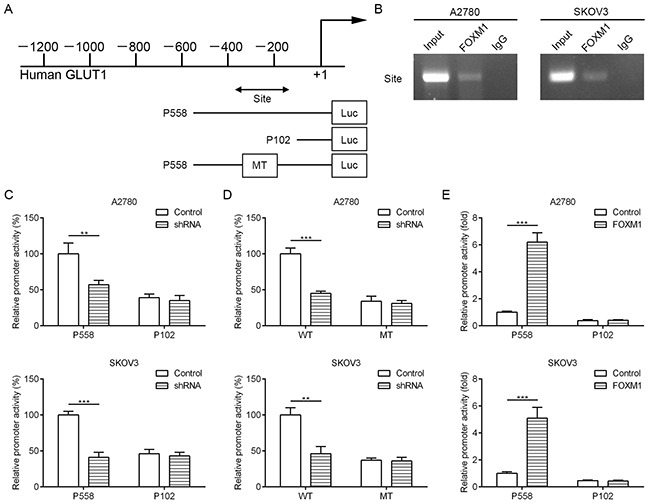
FOXM1 binds to human GLUT1 promoter and directly enhances its transcription **A.** a putative FOXM1-binding site in the GLUT1 promoter and construction of reporter plasmids. **B.** ChIP analysis of the GLUT1 promoter using antibodies against FOXM1 in A2780 and SKOV3 cells. **C.** the promoter activity of two truncated constructs was measured in A2780 and SKOV3 cells when cotransfected with the control plasmid or FOXM1 shRNA plasmid. **D.** the transcriptional activity of FOXM1 on GLUT1-luc wide type (WT) or mutants (MT) was analyzed by luciferase reporter assay in A2780 and SKOV3 cells. **E.** the promoter activity of two truncated constructs was measured in A2780 and SKOV3 cells when cotransfected with the control plasmid or pcDNA3.1-FOXM1 plasmid. Promoter activity was examined using a dual luciferase assay kit. The data represent three independent experiments, each bar represents mean ± SD. *P* values were calculated using a Student t-test (**P < 0.01, ***P < 0.001).

To dissect the molecular mechanism of the effects of FOXM1 on GLUT1 expression, we analyzed the sequences of GLUT1 promoter for the potential FOXM1-binding elements. Intriguingly, we identified a putative FOXM1-binding element in the GLUT1 promoter region (Figure 4A). To explore whether FOXM1 directly regulates GLUT1, we first performed ChIP assays in A2780 and SKOV3 cells. The results suggested that GLUT1 chromatins were specifically immunoprecipitated with antibody against FOXM1, compared with the IgG control (Figure 4B). Moreover, a series of reporter gene constructs based on the potential binding sites were generated (Figure 4A). These reporter constructs were cotransfected into A2780 and SKOV3 cells with FOXM1 shRNA, pcDNA3.1–FOXM1 or control vector. As shown in Figure 4C, knockdown of FOXM1 significantly decreased the GLUT1 promoter activity in the P558 construct, and altered expression of FOXM1 did not change the promoter activity in the P102 construct, which did not contain the potential FOXM1 binding site. We mutated the putative binding sites within the luciferase reporter constructs (Figure 4A). As shown in Figure 4D, knockdown of FOXM1 significantly reduced the activity of the WT (wild-type) pLuc-GLUT1 construct in A2780 and SKOV3 cells, and altered expression of FOXM1 did not change the activity of the MT (mutant) pLuc-GLUT1 construct. Additionally, FOXM1 overexpression markedly increased the GLUT1 promoter activity in the P558 construct, and altered expression of FOXM1 did not change the promoter activity in the P102 construct (Figure 4E). Collectively, these results support that FOXM1 is an authentic and direct transcriptional activator for GLUT1.

### FOXM1 is a transcriptional activator of HK2

**Figure 5:**
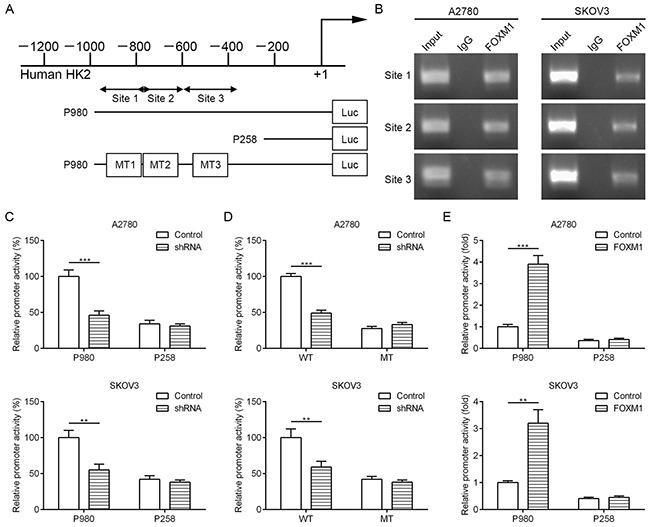
FOXM1 binds to human HK2 promoter and directly enhances its transcription **A.** putative FOXM1-binding sites in the HK2 promoter and construction of reporter plasmids. **B.** ChIP analysis of the HK2 promoter using antibodies against FOXM1 in A2780 and SKOV3 cells. **C.** the promoter activity of two truncated constructs was measured in A2780 and SKOV3 cells when cotransfected with the control plasmid or FOXM1 shRNA plasmid. **D.** the transcriptional activity of FOXM1 on HK2-luc wide type (WT) or mutants (MT) was analyzed by luciferase reporter assay in A2780 and SKOV3 cells. **E.** the promoter activity of two truncated constructs was measured in A2780 and SKOV3 cells when cotransfected with the control plasmid or pcDNA3.1-FOXM1 plasmid. Promoter activity was examined using a dual luciferase assay kit. The data represent three independent experiments, each bar represents mean ± SD. *P* values were calculated using a Student t-test (**P<0.01, ***P<0.001).

To dissect the molecular mechanism of the effects of FOXM1 on HK2 expression, we analyzed the sequences of HK2 promoters for the potential FOXM1-binding elements. Intriguingly, we identified three putative FOXM1-binding elements in the HK2 promoter region (Figure 5A). To explore whether FOXM1 directly regulates HK2, we first performed ChIP assays in A2780 and SKOV3 cells. The results suggested that HK2 chromatins were specifically immunoprecipitated with antibody against FOXM1, compared with the IgG control (Figure 5B). Moreover, a series of reporter gene constructs based on the potential binding sites were generated (Figure 5A). These reporter constructs were cotransfected into A2780 and SKOV3 cells with FOXM1 shRNA, pcDNA3.1–FOXM1 or control vector. As shown in Figure 5C, knockdown of FOXM1 significantly decreased the HK2 promoter activity in the P980 construct, and altered expression of FOXM1 did not change the promoter activity in the P258 construct, which did not contain the potential FOXM1 binding sites. We mutated the putative binding sites within the luciferase reporter constructs (Figure 5A). As shown in Figure 5D, knockdown of FOXM1 significantly reduced the activity of the WT (wild-type) pLuc-HK2 construct in A2780 and SKOV3 cells, and altered expression of FOXM1 did not change the activity of the MT (mutant) pLuc-HK2 construct. Additionally, FOXM1 overexpression markedly increased the HK2 promoter activity in the P980 construct, and altered expression of FOXM1 did not change the promoter activity in the P258 construct (Figure 5E). Collectively, these results support that FOXM1 is an authentic and direct transcriptional activator for HK2.

### Correlation of FOXM1, GLUT1 and HK2 expression in EOC patients

**Figure 6:**
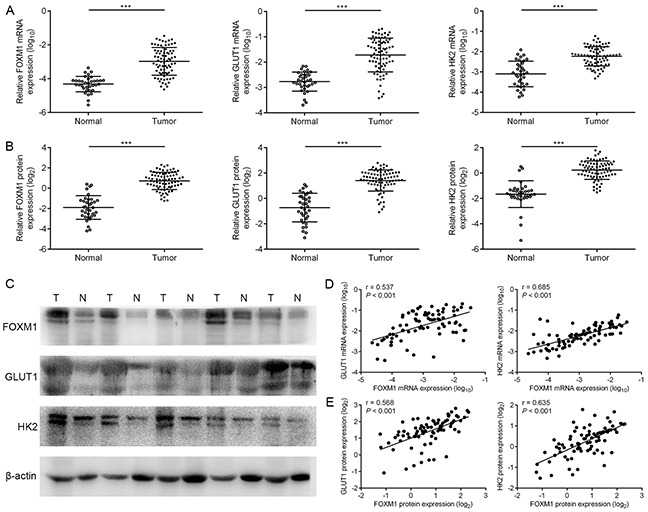
The coordinate expression of FOXM1, GLUT1 and HK2 in EOC tissues **A.** levels of FOXM1, GLUT1 and HK2 mRNA expressions in seventy-eight EOC (T) and thirty-five normal ovarian tissues (N) by qRT-PCR analysis. **B.** and **C.** levels of FOXM1, GLUT1 and HK2 protein expressions in seventy-eight EOC (T) and thirty-five normal ovarian tissues (N) by western blot analysis. The β-actin protein served as a loading control. **D.** an interrelationship between FOXM1, GLUT1 and HK2 mRNA levels in EOC tissues. Gene expression was determined relative to β-actin. **E.** an interrelationship between FOXM1, GLUT1 and HK2 protein levels in EOC tissues. Columns, mean of three independent experiments; bars, s.d. ***P < 0.001, Student's t-test.

To explore the role of FOXM1, GLUT1 and HK2 for ovarian tumorigenesis, we characterized their expression status in thirty-five human normal ovarian tissue samples and seventy-eight human EOC tissue samples. The mRNA and protein expressions of these three biomarkers were confirmed to be higher in EOC tissues than in ovarian normal tissues, respectively (Figure 6A-6C). These data demonstrated that FOXM1, GLUT1 and HK2 were aberrantly expressed in EOC patients, indicating that they may play important roles in the development and progression of EOC.

**Figure 7:**
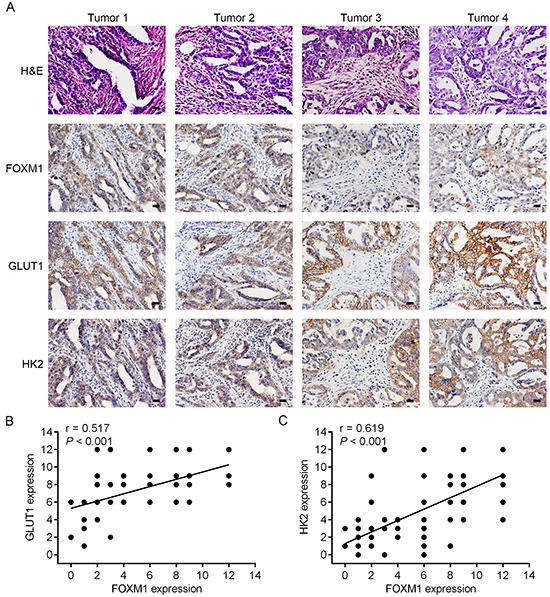
Concomitant expression of FOXM1, GLUT1 and HK2 in EOC patient specimens by immunohistochemical analysis **A.** representative staining of H&E, FOXM1, GLUT1 and HK2 of tumor sections from patients with EOC. All four of the tested specimens (Tumor 1-Tumor 4) showed a positive indication of FOXM1, GLUT1 and HK2 (Bar, 100 μm). **B.** correlation analysis of FOXM1 and GLUT1 expression in EOC tissues (n = 78, Pearson correlation coefficient). **C.** correlation analysis of FOXM1 and HK2 expression in EOC tissues (n = 78, Pearson correlation coefficient). Note that some of the dots on the graphs represent more than one specimen.

To test the clinical relevance of the above findings, we investigated the expression of FOXM1, GLUT1 and HK2 in EOC specimens. On comparing the mRNA expression levels of these three biomarkers, we observed that tumors exhibiting high FOXM1 mRNA expression also expressed elevated mRNA levels of GLUT1 and HK2, indicating that a positive correlation between FOXM1 mRNA and GLUT1 mRNA levels, and a positive correlation between FOXM1 mRNA and HK2 mRNA levels (Figure 6D). Also, in the same set of EOC specimens, we examined the protein levels of FOXM1, GLUT1 and HK2 by western blotting. Western blot analyses indicated that the observed levels of FOXM1 protein positively correlated with the levels of GLUT1 and HK2 protein in these tumors (Figure 6E). Of note, using the same EOC specimens, this was also independently confirmed by immunohistochemical analysis. We also found a significant linear correlation between FOXM1 and GLUT1, and between FOXM1 and HK2 in EOC specimens (Figure 7A-7C). These results support our finding that FOXM1 is strictly coexpressed with GLUT1 and HK2 in EOC and our findings in model systems find a close parallel in clinical samples.

## DISCUSSION

In the present study, we used two EOC cell lines with FOXM1 knockdown to systemically address the role of FOXM1 in aerobic glycolysis in EOC cells. First, the downregulation of FOXM1 by two different FOXM1 shRNAs decreased glycolysis in both A2780 and SKOV3 cells, suggesting a metabolic mechanism for tumor growth in EOC cells. Second, we found that FOXM1-silenced A2780 and SKOV3 cells had lower expression of GLUT1 and HK2 at both mRNA and protein levels compared to control cells. Third, FOXM1 bound directly to the promoter regions of GLUT1 and HK2 and regulated expression of GLUT1 and HK2 at the transcriptional level. Finally, we found that FOXM1 was concomitantly overexpressed with GLUT1 and HK2 in EOC specimens. These results clearly indicate that overexpression of FOXM1 promotes reprogramming of glucose metabolism at least in part through the transcriptional upregulation of GLUT1 and HK2 in EOC cells.

FOXM1 is well-known for its critical role in cell cycle progression by regulating the transition from G1 to S phase and G2 to M phase progression, as well as to mitosis [19, 33, 34]. Besides its essential roles in cell cycle regulation, FOXM1 also emerged as an oncogenic transcription factor with a high expression and functional impact in many types of cancer cells [23–32]. Our previous study had shown that overexpression of FOXM1 was associated with lymph node status and poor patient survival in EOC [31]. Thus far, there is no direct evidence reported to support that FOXM1 promotes reprogramming of glucose metabolism in EOC cells. Reprogramming of glucose metabolism is a hallmark in various tumor origins, and serves cancer cells in proliferation and survival through maintenance of biosynthesis and redox homeostasis [7–9]. To meet cancer cell energy needs, the glycolytic switch is associated with increased glucose uptake and accumulation of lactate. Several pieces of evidence suggest that lactate actively participates in angiogenesis and metastasis of several cancer types through the activation of several molecular pathways [35]. Furthermore, previous studies show that cancer cells with stimulated invasiveness get survival benefit from the glycolytic switch [36]. Cancer cells facilitate the metabolic shift to glycolysis to promote cancer progression, and this metabolic shift may be reversible and is partly due to aberrant regulation of glycolytic enzymes [37, 38]. Here we showed that knockdown of FOXM1 in EOC cells did not change the expression of most glycolytic enzymes except for GLUT1 and HK2. We found that FOXM1 positively regulated the transcriptions of GLUT1 and HK2 and promoted the aerobic glycolysis in EOC cells.

Given that GLUT1 and HK2 are critical glycolysis-related enzymes, and that their expressions are most significantly regulated by FOXM1, we reason that overexpression of FOXM1 likely plays a key role in the aerobic glycolysis and proliferation of EOC cells. The facilitative glucose transport protein GLUT1 has been shown to be closely related to ^18^F-FDG and glucose uptake in cancer cells, and its high expression in tumors has been associated with poor prognosis [39–42]. HK2 also plays a major role in aerobic glycolysis, catalyzing its first step and preventing glucose from entering the cell [43]. Previous studies have confirmed that HK2 expression was significantly higher in a variety of malignant tumors [43, 44]. In the present study, knockdown of FOXM1 downregulated GLUT1 and HK2 expression, resulting in an inhibition of aerobic glycolysis in EOC cells. A previous report showed that FOXM1 played important roles in reprogramming of glucose metabolism in pancreatic cancer via transcriptional regulation of LDHA expression [32]. Here we found that inhibition of FOXM1 expression by shRNA had no effect on LDHA expression in EOC cells, the mechanisms involved could be different in different types of cancer.

Given that FOXM1, GLUT1 and HK2 play instrumental roles in cell proliferation and aerobic glycolysis of cancer cells, we sought to determine the underlying mechanisms that may be responsible for coexpression of these three biomarkers. In this study, we confirmed that both GLUT1 and HK2 expression were significantly correlated with FOXM1 expression in both *in vitro* and *in vivo* experiments. We further investigated whether FOXM1, an oncogenic transcription factor, regulated GLUT1 and HK2 expression via transcription in EOC cells. We observed that FOXM1 bound directly to the GLUT1 and HK2 promoter regions to promote their transcription. To the best of our knowledge, this is the first study to describe a novel role of FOXM1 in regulation of GLUT1 and HK2 in cancer cells. In addition, we observed that FOXM1, GLUT1 and HK2 were significantly overexpressed in EOC tissues in comparison to those in normal tissues, respectively. Further statistical analysis indicated that there was a positive correlation between expression of FOXM1 and GLUT1, and a positive correlation between expression of FOXM1 and HK2 in EOC tissues. Thus, our findings reveal two novel FOXM1-GLUT1 and FOXM1-HK2 signaling pathways that play a critical role in promoting aerobic glycolysis in EOC cells.

In summary, our study here provides direct evidence that FOXM1 promotes reprogramming of glucose metabolism in EOC cells via activation of GLUT1 and HK2 transcription, which provides a new mechanisms for the Warburg effect. Our results suggest that targeting FOXM1 may result in further treatment avenues in the metabolic modulation of EOC and merit further investigation in future research.

## MATERIALS AND METHODS

### Collection of human tumors

This study was approved by the Ethics Committee of the Fourth Military Medical University. Thirty-five human normal ovarian tissue samples and seventy-eight human EOC tissue samples were obtained for diagnostic purposes with the consent of each patient between 2004 and 2007. Clinical data were obtained from clinical databases and tumors were staged according to International Federation of Gynecology and Obstetrics (FIGO) guidelines. None of the patients underwent chemotherapy or other adjuvant treatments before surgery.

### Cell culture and transfection

SKOV3 cells were obtained from American Type Culture Collection (ATCC) and were cultured in Dulbecco's modified Eagle's medium (Gibco BRL, Gaithersburg, MD). A2780 cells were obtained from Sigma-Aldrich Corporation (St. Louis, MO) and were cultured in RPMI 1640. All cells were cultured with 10% fetal bovine serum (Gibco BRL, Gaithersburg, MD), 100 U/mL penicillin (Invitrogen, Carlsbad, CA), and 100 mg/mL streptomycin, and were incubated at 37°C in 5% CO_2_ humidified air. Cells were cytogenetically tested and authenticated before being frozen. Transfections were performed with Lipofectamine 2000 reagent (Invitrogen, Carlsbad, CA) using 1–2 mg of expression vector/ml serum-free medium as described by the manufacturer. The coding regions of FOXM1 were inserted into pcDNA3.1 (Clontech, Mountain View, CA). A lentiviral vector carrying FOXM1 shRNA was used to silence FOXM1 in A2780 and SKOV3 cells, and stable clones were generated by puromycin selection.

### Quantitative real-time PCR

Total RNA isolation from cell lines and tissues was performed using Trizol (Invitrogen, Carlsbad, CA). A reverse transcription reaction was performed using a reverse transcription kit (Applied Biosystems, Foster City, CA). Quantitative real-time PCR (qRT-PCR) was performed on an ABI 7500 real-time system (Applied Biosystems, Foster City, CA) according to the manufacturer's protocol. Data were analyzed according to the comparative Ct method [45]. The β-actin was used as an internal control for each specific gene. Three independent experiments were performed to analyze the relative gene expression. Primer sequences are listed in Supplementary Table S1.

### Western blot analysis

Cells were harvested and homogenized with lysis buffer, and western immunoblotting was performed using standard procedures. Total protein was separated on 10% SDS-PAGE gels and transferred to PVDF membrane (Millipore, Bedford, MA). The membrane was blocked with 5% nonfat dry milk in TBS, then probed with the antibody against FOXM1 (Santa Cruz Biotechnology, Dallas, TX), GLUT1 (Abcam, Cambridge, MA), HK2 (Abcam, Cambridge, MA) and β-actin (Abcam, Cambridge, MA). After washing, horseradish peroxidase-conjugated anti-rabbit IgG (Santa Cruz Biotechnology, Dallas, TX) was used as a secondary antibody and incubated for 1 h at room temperature. Quantification of band intensity was performed using Image J software.

### Immunohistochemistry

The immunostaining technique was conducted as described previously [46]. The intensity of staining was scored as 0 (negative), 1 (weak), 2 (medium) or 3 (strong), while the extent of staining was scored as 0 (0% of cells stained), 1 (1–25%), 2 (26–50%), 3 (51–75%) or 4 (76–100%). The scores of each tumor sample were multiplied to give a final score of 0–12.

### Chromatin immunoprecipitation assay

Chromatin immunoprecipitation (ChIP) assays were performed in EOC cells following the protocol provided by the manufacturer (Millipore, Bedford, MA). Briefly, after cross-linking with formaldehyde at 1% final concentration for 10 min at 37°C and the reaction was quenched by addition of glycine to a final concentration of 0.125 M. The cells were lysed in SDS buffer and the pellet was resuspended in nuclei lysis buffer and sonicated. Immunoprecipitation was carried out with FOXM1 antibody (Santa Cruz Biotechnology, Dallas, TX). The PCR primer sequences for DNA fragments as parts of the targeted promoters are provided in Supplementary Table S2.

### Luciferase reporter assay

The luciferase assays were performed using a luciferase assay kit (Promega, Madison, WI) according to the manufacturer's protocol. Cells were plated in 24 well plates and transiently transfected with pGL3-GLUT1 or HK2 vector and Renillar luciferase reporter with FOXM1 shRNA, pcDNA3.1–FOXM1 or control vector using Lipofectamine 2000 (Invitrogen, Carlsbad, CA). Relative firefly luciferase activity was measured using a dual luciferase assay system (Promega, Madison, WI) 24 hours after transfection.

### Metabolic assays

Glucose uptake was measured using cell lysates with a glucose assay kit (Biovision, Milpitas, CA) according to the attached protocol. Glycolysis rate was measured by monitoring the conversion of 5-^3^H-glucose to ^3^H-H_2_O as described [47, 48]. Lactate production in the culture media of cells was detected by using a lactate assay kit (Biovision, Milpitas, CA) according to the manufacturer's instructions. Oxygen consumption in cells was examined by using the BD oxygen biosensor system (BD Biosciences, San Jose, CA) following the manufacturer's instruction.

### ^18^F-FDG micro-PET/CT imaging

Cells (5 × 10^6^) were implanted subcutaneously into the left flank of nude mice, and five mice were included in one experimental group. The xenograft-bearing mice were fasted overnight and anesthetized with 2% isoflurane. ^18^F-FDG of about 200 μCi was injected into the tail vein of each mouse. Sixty minutes after ^18^FDG injection, the PET/CT data acquisition procedure was performed on a micro-PET/CT system (Mediso, Boston, MA). All PET/CT images were processed and analyzed with Interview Fusion 1.0 (Mediso, Boston, MA) software.

### Xenograft model

BALB/C nude mice aged 4-6 weeks old were purchased from Shanghai Laboratory Animal Center (SLAC, Shanghai, China) and housed within a dedicated SPF facility at the Laboratory Animal Center of the Fourth Military Medical University. All studies were performed following guidelines approved by the Institutional Animal Ethics Committee of the Fourth Military Medical University. For tumor growth evaluation, A2780 cells (5 × 10^6^) were stably transfected with control shRNA or FOXM1 shRNA and subcutaneously injected into the left flank of nude mice. Tumor volumes were measured every 3 days with a caliper, and calculated using the formula V=length×width^2^/2. The mice were humanely killed on day 28, and subcutaneous tumors were surgically excised, weighed and photographed. Tumor tissues were sectioned and stained with hematoxylin-eosin (H&E) and Ki67 that is a mitotic marker. The expression levels of FOXM1, GLUT1 and HK2 were analyzed by qRT–PCR, western blotting and immunohistochemistry.

### Statistical analysis

All data were expressed as mean ± standard deviation (S.D.), and then processed using GraphPad Prism v5.0 software. A Student's t-test was performed to compare the differences between treated groups relative to their paired controls. Pearson correlation coefficient was used to measure the strength of the association between FOXM1, GLUT1 and HK2 expression levels. Values of *P* < 0.05 were considered significant.

## SUPPLEMENTARY TABLES


