# Supplementary material for: FOXM1 promotes reprogramming of glucose metabolism in epithelial ovarian cancer cells via activation of GLUT1 and HK2 transcription
Source: Oncotarget. 2016 Jun 16;7(30):47985–97. doi: 10.18632/oncotarget.10103 (PMC5216994; doi:10.18632/oncotarget.10103)
Supplement: Supplementary file 1 [file oncotarget-07-47985-s001.pdf]

FOXM1 promotes reprogramming of glucose metabolism in epithelial ovarian cancer cells via activation of GLUT1 and HK2 transcription

SUPPLEMENTARY TABLES

Supplementary Table S1: Quantitative real-time PCR primers used to amplify metabolic enzyme mRNAs

Gene	Primer sequence
AldoB	
Forward	5’-ATGCCACTCTCAACCTCAATGCTATC-3’
Reverse	5’-TTATTTTCTTGGGTGGGTATTCTGG-3’
Enolase	
Forward	5’-CTGATGCTGGAGTTGGATGG-3’
Reverse	5’-CCATTGATCACGTTGAAGGC-3’
FOXM1	
Forward	5’-CACCCCAGTGCCAACCGCTACTTG-3’
Reverse	5’-AAAGAGGAGCTATCCCCTCCTCAG-3’
GLUT1	
Forward	5’-CTTTGTGGCCTTCTTTGAAGT-3’
Reverse	5’-CCACACAGTTGCTCCACAT-3’
GLUT4	
Forward	5’-CTTCATCATTGGCATGGGTTT-3’
Reverse	5’-CGGGTTTCAGGCACTTTTAGG-3’
GAPDH	
Forward	5’-CAGCCTCAAGATCATCAGCA-3’
Reverse	5’-TGTGGTCATGAGTCCTTCCA-3’
G6PI	
Forward	5’-AGGCTG CTGCCACATAAGGT-3’
Reverse	5’-AGCGTCGTGAGAGGTCACTTG-3’
HK2	
Forward	5’-GATTTCACCAAGCGTGGACT-3’
Reverse	5’-CCACACCCACTGTCACTTTG-3’
LDHA	
Forward	5’-CAGCTTGGAGTTTGCAGTTAC-3’
Reverse	5’-TGATGGATCTCCAACATGG-3’
LDHB	
Forward	5’-CCTAGAGCTCACTAGTCACAG-3’
Reverse	5’-CTCCTGTGCAAAATGGCAAC-3’

(*Continued*)

Gene	Primer sequence
PFK-L	
Forward	5'-GGACAGGAAAGAGGAAGTGAC-3'
Reverse	5'-CGTAGATGAGGAAGACTTTGGC-3'
PFK-M	
Forward	5'-ATTCGGGCTGTGTTCTGG-3'
Reverse	5'-TGGCTAGGATTTTGAGGATGG-3'
PFK-P	
Forward	5'-CATCGACAATGATTTCTGCGG-3'
Reverse	5'-CCATCACCTCCAGAACGAAG-3'
PGK-1	
Forward	5'-CGGTAGTCCTTATGAGCC-3'
Reverse	5'-CATGAAAGCGGAGGTTCT-3'
PKM1	
Forward	5'-CTATCCTCTGGAGGCTGTGC-3'
Reverse	5'-CCATGAGGTCTGTGGAGTGA-3'
PKM2	
Forward	5'-GGGTTCGGAGGTTTGATG-3'
Reverse	5'-ACGGCGGTGGCTTCTGT-3'

Supplementary Table S2: Genes and primer sequences for ChIP assay

Gene	Primer sequence
GLUT1	
Forward	5'-TCCCAGAGGAGGTGATTT-3'
Reverse	5'-CTCGTAGGGTTGTAGAAAGAC-3'
HK2	
Forward	5'-TTGGGTGAGAAAGAGTTTGTATT-3'
Reverse	5'-TCTTTTTCTTTCCCAGTTGTCTC-3'
Forward	5'-AGGGTGAGTGGCAAGAAGGAATC-3'
Reverse	5'-CTCACCATAGTAAATCTACAGCG-3'
Forward	5'-CTCGCTGTAGATTTACTATGGTG-3'
Reverse	5'-CATCTGGACACACCTTCCTCTCT-3'
